# Protocol for a Systematic Review: Interventions for Anxiety in School‐Aged Children with Autism Spectrum Disorder (ASD): A Mixed‐Methods Systematic Review

**DOI:** 10.1002/CL2.217

**Published:** 2018-01-10

**Authors:** Petra Lietz, Julie Kos, Katherine Dix, Jenny Trevitt, Mirko Uljarevic, Elizabeth O'Grady

## BACKGROUND

### Description of the condition

Autism spectrum disorders (ASD) are a group of neurodevelopmental disorders. Children with ASD typically have difficulty with communication and social interaction, and may be overly dependent on routines, place extreme foci on items, and/or extremely dislike changes to their environment (Diagnostic and Statistical Manual of Mental‐5, American Psychiatric Association, 2013). These symptoms appear on a continuum, with some children experiencing only mild symptoms, while others experience quite severe symptomatology.

The reported prevalence of children diagnosed with ASD has increased over time. While the current rate of prevalence in the United States (US) is the same as the rate reported in 2012 (1 in 68 children), it is a significant jump from the 1 in 88 children reported in 2008; and the 1 in 150 children reported in 2002 (Autism Speaks, 2016; Centers for Disease control and Prevention, 2016). This growth in prevalence has also been seen in other countries. For example, the Survey of Disability, Ageing and Carers (SDAC) conducted by the Australian Bureau of Statistics (ABS) shows that in 2009, about 64,400 people had been diagnosed with ASD, and in 2012, this had increased to 115,400 (ABS, 2016). More recent population‐level data have been collected by the ABS (see ABS, 2016), however it is currently not publicly available. Where possible, up‐to‐date prevalence rates will be included in our review. In addition to increasing numbers, recent research shows that the number of students with ASD attending mainstream schools is also increasing (Zainal & Magiati, 2016). The exact reason for the increase in prevalence is unclear, but may be related to changes in the Diagnostic and Statistical Manual of Mental Disorders (DSM)^1^, as well as increased awareness and better recognition of less severe cases that were otherwise previously diagnosed as anxiety, bipolar, or other related disorder.

ASD often co‐occurs with other neurodevelopmental and neuropsychiatric disorders, including Attention‐Deficit/Hyperactivity Disorder (ADHD), learning difficulties, depression and anxiety. Anxiety symptoms have been noted in individuals with ASD since Leo Kanner and Hans Asperger first described this disorder more than 70 years ago (Lyons & Fitzgerald, 2007). Recent research continues to show that those with ASD exhibit significantly higher rates of anxiety symptoms when compared to (i) typically developing individuals (Bellini, 2006; Gadow et al., 2005; Lopata et al., 2010), and (ii) those with other disorders, including Down's Syndrome (Evans et al., 2005), Williams Syndrome (Rodgers et al., 2012), and Conduct Disorder (Green, Gilchrist, & Cox, 2000).

Anxiety is characterised by fear. Symptoms can include somatic complaints, such as stomach ache, headache, sleeplessness, and diarrhoea, as well as other symptoms including tiredness, irritability, and difficulty concentrating (Beyondblue, 2017). Some level of anxiety is normal. However, when the fear is persistent, excessive and interferes with one's ability to *function normally*, a diagnosis of an anxiety condition may be warranted.

Although the reported rate of anxiety for those with ASD varies widely (e.g., from 13% to 84%: van Steensel, Bogels, & Perrin, 2011), the majority of studies suggest that a more realistic estimate is 40 to 50 percent. A systematic review by van Steensel et al. (2011) supported this by reporting that 39.6% of the 2,121 individuals included in the reviewed studies met criteria for clinically elevated levels of anxiety.

The majority of studies undertaken in the area of anxiety and ASD have included very young children, older adolescents and adults. While fewer studies have been undertaken with school‐aged children, those studies that have been conducted suggest a high occurrence of anxiety in ASD populations (Ashburner et al., 2010; Gjevik et al., 2011; Lecavalier, 2006).

The prevalence of anxiety among school‐aged children is of particular concern considering that anxiety during this period has a negative impact on intellectual functioning and academic achievement, and broadly on a child's overall school‐functioning. School may present students with ASD particular cognitive, social and behavioural challenges that may increase levels of anxiety, and conversely, increased anxiety can impair school‐functioning. In addition, teachers tend to perceive students with ASD as having more difficulty with academic success and with anxiety than their typically developing peers (Ashburner et al., 2010).

Additional studies of children with an ASD have shown that anxiety negatively impacts a child's ability to participate in home, school and community settings, and effects child and family well‐being and quality of life above and beyond the core symptoms of ASD (Davis III, White, & Ollendick, 2014; Pellecchia et al., 2015).

Anxiety also has long term impacts. If left untreated, anxiety persists into adulthood (US Public Health Service, 2000), and can progress into other disorders, such as depression (Seligman & Ollendick, 1998). Moreover, chronic anxiety is related to reduced employment opportunities and social networks, and thus is associated with societal and economic burden (Davis et al., 2008; Velting et al., 2004).

Interventions and programs that aim to address anxiety and the challenges that school‐aged children with ASD face in educational environments, may improve their overall school‐functioning and later life‐outcomes.

Against this background, the need for accurate treatment of anxiety in school‐aged children with ASD is evident. Hence, the proposed systematic review aims to examine the effectiveness of interventions for anxiety, and anxiety related school‐functioning, including internalising and fear behaviours, for school‐aged children with ASD.

### Description of interventions

There are numerous interventions currently available for the treatment of anxiety in children and young people. The focus of this review will be on interventions designed to help a child's functioning in real‐world settings such as school and the home. Thus, studies assessing only the impact of pharmacological interventions will be excluded, while a study investigating the impact of cognitive behaviour therapy (CBT) on academic performance would be included. We note that research indicates CBT is useful for treating anxiety disorders, but less is known about its efficacy in treating anxiety with ASD populations (Nadeau et al., 2011).

An example of a study to be included is:

Wood, J. J., Drahota, A., Sze, K., Har, K., Chiu, A., & Langer, D. A. (2009). Cognitive behavioral therapy for anxiety in children with autism spectrum disorders: A randomized, controlled trial. *The Journal of Child Psychology and Psychiatry*, 50 (3), 224–234.

*This study tested a modular cognitive behavioral therapy (CBT) program for children with ASD and anxiety. A standard CBT program was augmented with multiple treatment components designed to accommodate or remediate the social and adaptive skill deficits of children with ASD that could pose barriers to anxiety reduction. Forty children (7‐11 years old) were randomly assigned to 16 sessions of CBT or a 3‐month waitlist (36 completed treatment or waitlist). Therapists worked with individual families. The CBT model emphasized behavioral experimentation, parent‐training, and school consultation. Independent evaluators blind to treatment condition conducted structured diagnostic interviews and parents and children completed anxiety symptom checklists at baseline and post‐treatment/post‐waitlist. Results showed that 78.5% of the CBT group met Clinical Global Impressions‐Improvement scale criteria for positive treatment response at post‐treatment, as compared to only 8.7% of the waitlist group. CBT also outperformed the waitlist on diagnostic outcomes and parent reports of child anxiety, but not children's self‐reports. Treatment gains were maintained at 3‐month follow‐up. Conclusions: The CBT manual employed in this study is one of the first adaptations of an evidence‐based treatment for children with autism spectrum disorders.*

### Importance of this review

Since children spend a significant portion of their day at school, teachers and clinicians working in the education sector have significant responsibility for recognising signs of ASD and anxiety, and in implementing interventions and supports that are evidence‐based and tailored to the needs of the child. Further, decision‐making regarding treatment should be informed by the latest evidence available. However, the sheer volume of published research, and the different aims, foci and methodology of those studies, makes evidence‐based practice extremely difficult for professionals, including for those working in the education sector. The current review will provide a much needed source of valuable information for various professionals.

Finally, a number of narrative and systematic reviews on various aspects of anxiety in ASD have been published in the last ten years. These reviews have covered phenomenology and prevalence of anxiety (White et al., 2009; MacNail, Lopez, & Minnes, 2009; van Steensel et al., 2010), assessment (Wigham, & McConachie, 2014; Lecavalier et al., 2014) and treatment (Johnco & Starch, 2o15; Kreslins, Robertson, & Melvile, 2015; Ung et al., 2015; Vasa et al., 2014; Sukhodolsky et al., 2013). However, none of the reviews published thus far have: (i) focused specifically on school‐aged children with ASD; (ii) covered the range of available treatments, but instead focussed only on specific treatments, such as, for example, Cognitive Behaviour Therapy or psychosocial treatments; (iii) explored mediators and moderators of treatment outcomes; (iv) provided practical guidance for education professionals and parents to enable increased use of evidence‐based treatments in their everyday practice.

## OBJECTIVES

This review aims to synthesise evidence about interventions to reduce anxiety symptoms in school‐aged children with ASD. While clinical studies will not be excluded per se, this review seeks to move beyond interventions that are relevant only for clinical practice and care in clinical settings and prioritise studies that draw out implications for school‐aged children that will help their functioning in real‐world settings such as school and the home.

To achieve this aim, the review will employ a mixed‐methods systematic review (JBI, 2014b) which can accommodate the anticipated diverse types of available studies. These studies are likely to use quantitative methods such as quasi‐experimental, mixed‐methods randomised control trial approaches as well as qualitative methods such as action‐research and case‐study designs.

This systematic review aims to address the following research question.

1. What is the relative effectiveness of interventions for managing anxiety of school‐aged children with ASD that have been used in school, family, and clinical settings?

In the process, this review will also identify:

- the interventions used for managing anxiety of school‐aged children with ASD in school, family, and clinical settings;
- the sources of variability in response to the intervention. For example, (i) what works for whom and how? (ii) under what circumstances are interventions efficacious or otherwise? (iii) are there sub‐groups that the intervention impacts differentially (e.g., race, age, socio‐economic status, academic achievement)?
- the evidence‐based practices that school staff, parents, and other professionals can employ to mitigate anxiety‐related symptoms in school‐aged children with ASD.

Results of the review are intended to inform professionals working in the education sector and parents, but may also inform policy makers in this sector.

## METHODOLOGY

Given that the focus is on a) interventions that address anxiety and school‐related functioning in school settings or family contexts and b) the intention for results of the review to be of direct relevance to policy makers and practitioners, it is anticipated that the studies relevant to this review will have employed various methodologies, not only quantitative but also qualitative methods. Therefore, a mixed‐methods systematic review (JBI, 2014b) is proposed as the appropriate methodology for this work.

More specifically, the mixed‐methods systematic review will follow the steps recommended by Sandelowski, Leeman, Knafl and Crandel (2012) with two separate syntheses conducted first, one resulting in a synthesis of quantitative studies and one resulting in a synthesis of qualitative studies. In the current review, the quantitative studies are proposed to be synthesised by way of meta‐analysis while the qualitative studies are proposed to be synthesised by way of meta‐aggregation (Lookwood, Munn & Porritt, 2015).

In the final step, the results of the two separate syntheses will be integrated by way of an aggregative mixed‐method synthesis. In order to integrate the results of the two syntheses, the results of the quantitative synthesis are translated into qualitative statements through Bayesian conversion. This is considered preferable to the translation of qualitative into quantitative statements as problems arise when attempting to convert verbal counts (e.g. “many”, “few”) into quantities (JBI, 2014b). The mixed‐methods approach to this systematic review is illustrated in Figure 1.

**Figure 1:**
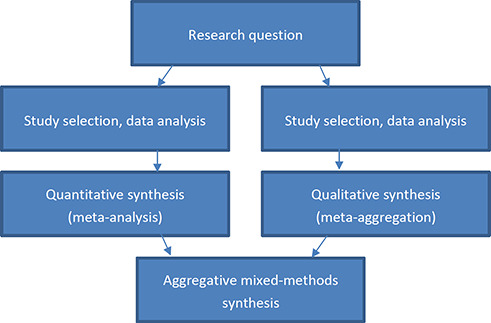
Steps in the proposed mixed‐methods review (JBI, 2014b, p. 10)

### Criteria for Inclusion and Exclusion of Studies in the Review

#### Population

The target population for the review is school‐aged children (5 to 18 years old) diagnosed with an ASD (inclusive of autism, ASD, Autistic Disorder, Asperger's Disorder, Asperger Syndrome, atypical autism, PDD‐NOS) by a professional eligible to diagnose these conditions, *and also* experiencing anxiety symptoms or a diagnosis of an anxiety disorder provided by a professional eligible to diagnose such conditions. While a formal diagnosis of an ASD will be required for inclusion, anxiety symptoms will not have to be formally diagnosed. Therefore, we will include anxiety symptoms such as internalising behaviours and fear in the review. The studies can occur in schools or out‐of‐school settings (e.g. home, larger community) or clinical settings, as long as the intervention/s is designed to improve outcomes in real‐world settings.

If studies include a sample of children in the target population as well as other children (e.g., the general population) and the findings are separated for the ASD sub‐group, the study will be included in the review whereby the type of ASD diagnosed will not matter. In contrast, if the study findings are not reported separately – for example – for children with ASD and ADHD are combined for analysis, the study will be excluded from the review as the impact of the intervention on only the ASD sample will be impossible to derive.

No restrictions will be imposed in terms of background variables such as socio‐economic status, or profiles of children with ASD ‐ for example with respect to characteristics such as IQ or ASD severity/classification. However, where possible, the mediating or moderating influence of these variables on the treatment outcome(s) will be explored.

The focus is on school‐aged children rather than earlier intervention since diagnosis of children with ASD who function at a relatively higher level often does not occur until primary school (Fombonne, 2003). To be included in the review, either all participants in a study have to be of school age or a majority of participants have to be of school age.

#### Types of Interventions

Rotheram‐Borus et al. (2012) proposed that all existing interventions for anxiety incorporate one or more of the following seven elements: (i) psychoeducation, (ii) exposure, (iii) cognitive restructuring, (iv) parent training or parent psychoeducation, (v) relaxation, (vi) modelling, and (vii) self‐monitoring. This review will include all treatments for anxiety for 5‐18 year olds with ASD in schools, families or in clinical settings that encompass at least one of these elements. As such, studies which focus solely on pharmacological interventions (e.g., selective serotonin reuptake inhibitors) will be excluded from the review. Where studies occur in a clinical setting, we will seek to draw out implications for real world settings such as the family and school.

All forms of assessing children are eligible for inclusion in the review, including diagnostic interviews, behavioural and physiological monitoring, as well as ratings scales ‐ irrespective of the informant (e.g., student, parent, teacher).

Two examples of included studies are provided for illustrative purposes.

Chalfant, A. M., Rapee, R., & Carroll, L. (2006). Treating anxiety disorders in children with high functioning autism spectrum disorders: A controlled trial. *Journal of Autism and Developmental Disorders*, 37 (10), 1842‐1857.

*A family‐based, cognitive behavioural treatment for anxiety in 47 children with comorbid anxiety disorders and High Functioning Autism Spectrum Disorder (HFA) was evaluated. Treatment involved 12 weekly group sessions and was compared with a waiting list condition. Changes between pre‐ and post‐treatment were examined using clinical interviews as well as child‐, parent‐ and teacher‐report measures. Following treatment, 71.4% of the treated participants no longer fulfilled diagnostic criteria for an anxiety disorder. Comparisons between the two conditions indicated significant reductions in anxiety symptoms as measured by self‐report, parent report and teacher report. Discussion focuses on the implications for the use of cognitive behaviour therapy with HFA children, for theory of mind research and for further research on the treatment components.*

Wood, J. J., Drahota, A., Sze, K., Har, K., Chiu, A., & Langer, D. A. (2009). Cognitive behavioral therapy for anxiety in children with autism spectrum disorders: A randomized, controlled trial. *The Journal of Child Psychology and Psychiatry*, 50 (3), 224–234.

*Background: Children with autism spectrum disorders often present with comorbid anxiety disorders that cause significant functional impairment. This study tested a modular cognitive behavioral therapy (CBT) program for children with this profile. A standard CBT program was augmented with multiple treatment components designed to accommodate or remediate the social and adaptive skill deficits of children with ASD that could pose barriers to anxiety reduction. Method: Forty children (7‐11 years old) were randomly assigned to 16 sessions of CBT or a 3‐month waitlist (36 completed treatment or waitlist). Therapists worked with individual families. The CBT model emphasized behavioral experimentation, parent‐training, and school consultation. Independent evaluators blind to treatment condition conducted structured diagnostic interviews and parents and children completed anxiety symptom checklists at baseline and post‐treatment/post‐waitlist. Results: In intent‐to‐treat analyses, 78.5% of the CBT group met Clinical Global Impressions‐Improvement scale criteria for positive treatment response at post‐treatment, as compared to only 8.7% of the waitlist group. CBT also outperformed the waitlist on diagnostic outcomes and parent reports of child anxiety, but not children's self‐reports. Treatment gains were maintained at 3‐month follow‐up. Conclusions: The CBT manual employed in this study is one of the first adaptations of an evidence‐based treatment for children with autism spectrum disorders. Remission of anxiety disorders appears to be an achievable goal among high‐functioning children with autism.*

An example of an excluded study, due to it being a pharmacological treatment, is provided.

Couturier, J., & Nicolson, R. (2002). A retrospective assessment of Citalopram in children and adolescents with Pervasive Developmental Disorders. *Journal of Child and Adolescent Psychopharmacology*, 12(3), 243‐8.

*Although selective serotonin reuptake inhibitors have been used to treat symptoms of aggression and anxiety in children and adolescents with pervasive developmental disorders (PDDs), there are no published reports of the use of citalopram in this population. The purpose of this study was to examine the benefits and adverse effects of citalopram in a group of children and adolescents with PDDs. Target behaviors included aggression, anxiety, stereotypies, and preoccupations. Seventeen patients with PDDs (14 with autistic disorder, three with Asperger's disorder) (mean age = 9.4 +/‐ 2.9 years; range 4‐15 years) were treated with citalopram for at least 2 months (mean duration of treatment = 7.4 +/‐ 5.3 months; range 1‐15 months). Treatment was initiated at a low dose (5 mg daily) and was increased by 5 mg weekly as tolerated and as necessary. The mean final dose was 19.7 +/‐ 7.8 mg (range 5‐40 mg). Outcome was based on a consensus between clinician and parents, using the Improvement item of the Clinical Global Impressions Scale as a guide. Ten (59%) children were judged to be much improved or very much improved regarding target behaviors. Core symptoms of PDDs (social interactions, communication) did not show clinically significant improvement. Citalopram was generally well tolerated, although four patients developed treatment‐limiting adverse effects: two with increased agitation, one with insomnia, and one with possible tics. The results of this case series suggest that citalopram has beneficial effects on some interfering behaviors associated with PDDs with few adverse effects. Controlled trials are warranted.*

### Outcomes

The aim is to review interventions that address anxiety symptoms in school‐aged children with a formal diagnosis of ASD in school, home and/or clinical settings. Measurement of anxiety (and related terms) should be undertaken using valid and reliable approaches such as screening instruments, observational ratings, and behavioural checklists. This review will include studies which refer to the variety of school‐functioning related outcomes that are often associated with anxiety, including social or emotional skills in the school or family setting, school engagement, learning outcomes and/or academic achievement.

### Types of Study Designs

Study designs to be included can be quantitative (e.g. descriptive, correlational, quasi‐experimental and experimental) as well as qualitative (e.g. ethnographic, narrative, phenomenological, grounded theory and case study) in kind.

As studies employing a qualitative design are less likely to employ some form of valid and reliable measurement of anxiety than quantitative studies, more of the latter than the former study designs are expected to be included in the review. It should also be noted that while single subject case‐studies often lack generalisability and tend not to use standardised assessments and outcome measures making comparability to other studies difficult, examples of this work are important (e.g., Beretvas & Chung, 2008; Ganz et al., 2012; Hedges, Pustejovsky, & Shadish, 2012; Shadish, Hedges, & Pustejovsky, 2014). Such designs are often published in psychology and in educational research.

Three examples of included studies are provided for illustrative purposes.

Clarke, C.D. (2012). An evaluation of a brief school‐based cognitive behavioural therapy programme for children with ASD (Doctoral thesis, University of London, England). Retrieved from http://ethos.bl.uk/OrderDetails.do?uin=uk.bl.ethos.573003

*Autistic Spectrum Disorder (ASD) is characterised by difficulties with social interactions, communication and rigid I stereotyped behaviours, with a prevalence of around 1% within the population. Research has shown that children with ASD also have heightened feelings of anxiety compared to typically developing peers, particularity with social anxiety. Cognitive Behavioural Therapy (CBT) has empirical evidence that demonstrates its efficacy in supporting children with ASD to manage their anxiety. However, these studies have only shown improvements in the children's anxiety using standardised questionnaires. As such, it is difficult to infer whether the gains made using CBT are long‐term, or whether it leads to a qualitative improvement in children's interactions with their community. Typically, CBT is typically delivered by Child and Adolescent Mental Health Services, which can be inaccessible to some children and their families. This study employed a mixed methods approach to understand the effectiveness of a six week, group administered, secondary school‐based CBT programme. 28 children took part in the research, with 14 in the treatment‐as‐usual group and 14 in the experimental group. All children completed the Wechsler Abbreviated Scale of Intelligence, the Social Responsiveness Scale, Spence Children's Anxiety Scale ‐ parent and child versions (SCAS‐P/C), the Coping Scale for Children and Youth (CSCY). Qualitative data was also collected through parent and child interviews using a semi‐structured technique. Post‐intervention data consisted of the SCAS‐P/C and the CSCY and further parent and child interviews. Follow‐up measures were taken six to eight weeks after post‐intervention using the SCAS‐Parent and child versions and the CSCY. Results suggest children who took part in the intervention had reduced levels of anxiety compared to the TaU group, both at post ‐ intervention and follow‐up. However, these improvements were not at a clinically significant level. Interview data, analysed using Thematic Analysis, provided unique insight into the process of cognitive change, the nature of anxiety in children with ASD and highlighted potential barriers to change for these children. Furthermore, the parents identified a lack of post diagnostic support and the view of their child's constantly changing profile of needs. The results are related to their implications for the professional educational psychologist, who is considered to be well placed to respond to the identified needs of this group and to implement CBT programmes in schools. Methodological issues and weaknesses are discussed as well as implications for further study.*

Reaven, J., Blakeley‐Smith, A., Culhane‐Shelburne, K., & Hepburn, S. (2012). Group cognitive behavior therapy for children with high‐functioning autism spectrum disorders and anxiety: A randomized trial. *Journal of Child Psychology and Psychiatry*, 53 (4), 410‐419. DOI: 10.1111/j.1469‐7610.2011.02486.x

*Background: Children with high‐functioning autism spectrum disorders (ASD) are at high risk for developing significant anxiety. Anxiety can adversely impact functioning across school, home and community environments. Cognitive behavioral therapies (CBT) are frequently used with success for children with anxiety symptoms. Modified CBT interventions for anxiety in children with ASD have also yielded promising results. Methods: Fifty children with high‐functioning ASD and anxiety were randomized to group CBT or treatment‐as‐usual (TAU) for 12 weeks. Independent clinical evaluators, blind to condition, completed structured interviews (Anxiety Disorders Interview Schedule‐Parent Version; ADIS‐P) pre‐ and post‐intervention condition. Results: Forty‐seven children completed either the CBT or TAU condition. Results indicated markedly better outcomes for the CBT group. Significant differences by group were noted in Clinician Severity Ratings, diagnostic status, and clinician ratings of global improvement. In the intent‐to‐treat sample, 10 of 20 children (50%) in the CBT group had a clinically meaningful positive treatment response, compared to 2 of 23 children (8.7%) in the TAU group. Conclusions: Initial results from this randomized, designed treatment study suggest that a group CBT intervention specifically developed for children with ASD may be effective in decreasing anxiety. Limitations of this study include small sample size, lack of an attention control group, and use of outcome measures normed with typically developing children.*

Sofronoff, K., Attwood, T., & Hinton, S. (2005). A randomised controlled trial of a CBT intervention for anxiety in children with Asperger syndrome. *Journal of Child Psychology and Psychiatry*, 46 (11), 1152‐1160.

*Background: The aim of the study was to evaluate the effectiveness of a brief CBT intervention for anxiety with children diagnosed with Asperger syndrome (AS). A second interest was to evaluate whether more intensive parent involvement would increase the child's ability to manage anxiety outside of the clinic setting. Methods: Seventy‐one children aged ten to twelve years were recruited to participate in the anxiety programme. All children were diagnosed with AS and the presence of anxiety symptoms was accepted on parent report via brief interview. Children were randomly assigned to one of three conditions: intervention for child only, intervention for child and parent, wait‐list control. Results: The two intervention groups demonstrated significant decreases in parent‐reported anxiety symptoms at follow‐up and a significant increase in the child's ability to generate positive strategies in an anxiety‐provoking situation. There were a number of significant differences between the two interventions to suggest parent involvement as beneficial. Conclusions: The sample of children with AS in this study presented with a profile of anxiety similar to a sample of clinically diagnosed anxious children. The intervention was endorsed by parents as a useful programme for children diagnosed with Asperger syndrome and exhibiting anxiety symptoms, and active parent involvement enhanced the usefulness of the programme. Limitations of the study and future research are discussed.*

The following study could be included although only if it were possible to separate the data for school‐aged children as some of the subjects are in pre‐school. However, it illustrates the type of school‐family collaborative treatments that will be considered for inclusion in the review.

Ruble, L. A., Dalrymple, N. J., & Mcgrew, J. H. (2010). The effects of consultation on individualized education program (IEP) Outcomes for young children with autism: The Collaborative Model for Promoting Competence and Success. *Journal of Early Intervention*, 32(4), 286‐301.

*The effects of a teacher consultation intervention were examined—namely, the collaborative model for promoting competence and success (COMPASS), which was designed to improve objectives of individualized education programs for children with autism. The intervention consists of an initial parent–teacher consultation, followed by four teacher consultations across the school year. Thirty‐five teachers and a randomly selected child with autism (M age = 6.1 years) from each classroom participated. Compared to the non‐intervention teacher–child dyads, the intervention teacher–child dyads showed improvements in individualized education program objectives, with a large effect size (d = 1.51).*

We expect a portion of the studies will be single case designs. Two examples of potential studies with a single‐case study methodology are provided below.

Camacho, R., Anderson, A., Moore, D. W., & Furlonger, B. (2014). Conducting a function‐based intervention in a school setting to reduce inappropriate behaviour of a child with autism. *Behaviour Change*, 31(1), 65‐77.

*Although function‐based interventions have been shown to be effective, the methods utilised to carry out functional behaviour assessments (FBA) have practical limitations. This study explored the relative utility and feasibility of three FBA methods in a school setting to inform a function‐based intervention to reduce problem behaviour in a boy with autism. The study consisted of: (1) indirect and direct assessments, (2) a modified functional analysis, and (3) the intervention. New video technology, Behavior Capture, was trialled to facilitate data collection in the classroom. All methods contributed to identifying the function of the problematic behaviour, though only the functional analysis provided conclusive results. A peer‐mediated intervention based on these findings conducted in the school playground reduced the problem behaviours. All FBA methods could be applied in the school setting and provided useful information. Novel technology was helpful in facilitating data collection. A naturalistic intervention was successful in reducing problem behaviours and increasing play skills.*

Vanderhoek, A., & Fielding‐Barnsley, R. (2004). Developing unique social stories as a behavioural intervention for an eight‐year old boy with Asperger Syndrome [online]. In B. Bartlett, F. Bryer, and D. Roebuck (Eds.), *Educating: Weaving Research into Practice: Volume 3* (pp. 194‐210). Nathan, Qld: Griffith University, School of Cognition, Language and Special Education.

*This case study examined the effectiveness of social story interventions for an eight‐year‐old Chinese boy diagnosed with mild Asperger's disorder in an international school in Hong Kong. A personalised approach based on Carol Gray's (1994) Social Story Handbook was utilised. Social stories focus on teaching children with ASD the social cues and behaviours they need to know to interact with others in a socially appropriate manner. Specifically the following behaviours were targeted: calling out to the teacher, laughing inappropriately, repeating what the teacher said and frequent visits to the toilet. Pre‐intervention observations were made over a one week period followed by a two‐week intervention which concluded with post‐intervention observations over a one‐week period. After this brief intervention promising results were obtained particularly in calling out and inappropriate laughter. The learning support teacher who initiated this intervention has been successful in transferring these skills to the classroom teacher who continues to use the social stories on a daily basis.*

It should be noted that established criteria will be used to assess the quality of the study design and resulting evidence which are appropriate to the design of the original primary study (e.g., Joanna Briggs Institute, 2014). Rather than omitting studies from the body of materials to be reviewed, the quality evaluation will be used to weight the findings from different studies.

### Search Strategy for Identification of Relevant Studies

Our search strategy is designed to identify published as well as unpublished literature via bibliographic databases, grey literature sources, selected websites, web searching, research registers and by manually searching targeted journals and reference lists. We will also attempt to contact key researchers in the field in order to identify any additional ongoing or unpublished studies.

#### Publication Date Range

Unless otherwise specified, our searches will be limited to a publication date range of 1996 to 2017. We selected 1996 as the earliest publication date in order to narrow the scope of interventions to current approaches used in the last 20 years. Given the development of understanding in this field, we believe that interventions before this date would be less progressive in their approach.

#### Other Criteria

The searches in our selected sources will not be restricted by geography, language, publication type or by publication status. However the selected sources are focused on the English language in keeping with our database subscriptions and the primary language of the authors.

#### Search terms

The following PICO (i.e. population, intervention, comparison and outcomes) concepts will form the basis of the search strategy.

Population:
School‐aged children (e.g., students, children, child, adolescents, preadolescents, youth, youths, pupils, teenagers, young people, young person, boys, girls) with ASD (e.g., Asperger Syndrome, Autism, ASD, Autistic Disorder, Asperger's Disorder, atypical autism, childhood autism, pervasive development disorders, PDD‐NOS, PDD‐unspecified).
AND
Outcomes: Anxiety (e.g., anxiety, anxious, internalise, internalisation, fear)
AND
Intervention: (e.g., intervention, treatment, therapy, psychotherapy, evaluation, outcome, program, trials, experimental, control group, random, best practice, evidence based)

It should be noted that we purposely have not specified a comparison as the review question is broad in nature. In such instances, the JBI (2013) suggest that a comparison statement may be inappropriate or undesirable, especially where multiple interventions may exist. In general, though, treatment as specified in the interventions will be compared to no treatment (e.g. waiting list).

Note that in the Intervention concept, we have included both intervention and study design terms. We were concerned that the use of the intervention concept may limit the search too narrowly particularly within the social, behavioural and educational databases. By including the alternative study design terms, we expect to maximise our chance of capturing the full range of various interventions that have been studied. However where this broad concept search results in an impractically large number of results and/or a significant reduction in the precision of the results, the search strategy will be limited to include the core, intervention terms: *intervention, treatment, therapy, psychotherapy.*

Our general search statement, set out below, will be customised to the available search strategies within the bibliographic databases and other sources that we search. Consideration will be given to available fields, limiters and subject indices as well as to other search features.

With the exception of Scopus database which does not have a controlled vocabulary, the subject indices for each of our bibliographic databases will be consulted in order to identify any additional, relevant search terms, beyond those already identified in our general search strategy. Various combinations of free text and/or controlled vocabulary terms will be used for each database. While trying to keep the search strategies as broad as possible to ensure relevant studies are not missed, the search strategy may need to be limited, to ensure a more manageable and precise set of results.

In searching the our sources beyond the bibliographic databases, the complexity of search features found in the databases may not always be available in the other sources and so simpler search strategies will be applied as needed.

This is our general search strategy that will guide our customised searches. All terms may not be included in every search statement:

ASD OR Asperger* OR autis* OR Pervasive Developmental Disorder OR PDD NOS OR PDD unspecified
AND
Anxiety OR anxious OR internali* OR fear
AND
Student OR child* OR adolescen* OR preadolescen* OR pre adolescen* OR youth OR teen* OR teen age* OR young people OR young person OR boy OR girl
AND
Intervention OR treatment OR therap* OR psychotherap* OR evaluation OR outcome OR program OR trial* OR experimental OR control group OR random* OR best practi* or evidence based

In this general search statement, the * symbol is used to indicate where our search will be designed to cover variations in the root of the word. This general statement assumes plural variations, requiring just the letter ‘s’, will be automatically searched.

#### Bibliographic Databases for Searching

The following bibliographic databases will be electronically searched for studies that match our inclusion criteria:

- Academic Search Complete (EBSCO)
- A+Education (Informit).
- British Education Index (EBSCO).
- CBCA Complete (Proquest)
- CINAHL (EBSCO).
- Education Research Complete (EBSCO)
- EMBASE (Elsevier)
- ERIC (EBSCO).
- PsycINFO (EBSCO).
- PubMed
- SCOPUS (Elsevier – complete search of Social Sciences/Humanities/Neuroscience collection)
- SocINDEX (EBSCO)

See Table 3 for customised search statements that will be applied to the bibliographic databases listed above.

#### Other Sources for Searching

##### Google

Google will be used to identify grey literature from websites in both government and organisation domains. Our general search statement will be customised to the Google Advanced Search. The searches will be limited to ‘Show the most relevant results’ and limited to the relevant date range. The search statements will be:

1. *anxiety intervention (autism OR asperger) OR (children OR adolescent) ‐pubmed site:gov filetype:pdf*
2. *anxiety intervention (autism OR asperger) OR (children OR adolescent) ‐pubmed site:org filetype:pdf*

##### OpenGrey (European)

OpenGrey will be used to identify relevant European grey literature. The search statement will be:

(ASD OR Asperger* OR autis* OR Pervasive Developmental Disorder* OR PDD NOS OR PDD unspecified) AND (Anxiety OR anxious OR internali* OR fear) AND (Student OR child* OR adolescen* OR preadolescen* OR pre adolescen* OR youth OR teen* OR teen age* OR young people OR young person OR boy OR girl)

##### Institutional Repositories

We will search the ‘Contents’ of the Directory of Open Access Repositories (OpenDOAR) to identify research papers from institutional repositories. Our general search statement will be customised to fit with the available search fields in the OpenDOAR Google Custom Search. Our search statement will be:

*autism OR Asperger anxiety intervention children OR adolescent ‐pubmed*

**Theses**

- Networked Digital Library of Theses and Dissertations – a customised search strategy will be used and the search will be limited by available population tags:
*(Asperger OR autism) AND Anxiety AND intervention*
- WorldCat – a customised search strategy will be used and the search will be limited by *Thesis/dissertation:*
*Asperger OR autism anxiety intervention*
- American Doctoral Dissertations (EBSCO) – our full search statement will be used.

##### Conference Proceedings

In addition to conference proceedings and papers indexed in our selected databases, we will identify conference literature via a search on SCOPUS which is a multidisciplinary database. This search will be limited to conference papers in the collections other than the Social Sciences, Humanities or Neuroscience as these collections will be completely searched as described in the section headed *Bibliographic Databases for Searching –* see Scopus.

##### Research Reviews

In designing the search for each of the following sources, consideration will be given to the size of the resource and whether the full search strategy is necessary or even possible to apply:

- Campbell Library
- Cochrane Central Register of Controlled Trials (CENTRAL)
- The JBI Database of Systematic Reviews and Implementation Reports.
- Database of Promoting Health Effectiveness Reviews (DoPHER)
- Evidence for Policy and Practice Information and Co‐ordinating Centre (EPPI‐Centre)
- Cochrane Database of Systematic Reviews
- PROSPERO International prospective register of systematic reviews

##### Targeted Searches of Selected Websites

We will browse or search the websites of selected agencies, research centres and professional associations including the following:

Agency for Heathcare Research and Quality
The Association for Science in Autism
Autism Consortium
Autism CRC
Autism Research Institute
Autism Speaks
Autism Spectrum Australia (Aspect)
Center for Autism Research
Centers for Disease Control and Prevention – Autism Spectrum Disorder (ASD)
Global Research in Autism and Neurodevelopment
Interactive Autism Research
The National Professional Development Center on ASD
National Institute of Health Care Excellence (NICE)
Research Autism
Simons Foundation Autism Research Initiative
World Health Organisation

##### Citations Searching

We will conduct forward citation searching on the studies identified for analysis, using SCOPUS database.

##### Manual Searching

We will search the reference lists of previously published reviews and meta‐analyses that we identify as well as the reference lists of each of the studies identified for our analysis.

##### Current literature

After our initial search, we will set up alerts in Google Scholar and, where possible, in the bibliographic databases, in order to identify any new literature within the time of our study. We will also manually scan the table of contents for the most current journal issues of our key journal titles.

##### Colleagues

We will identify and attempt to contact key researchers, seeking details of any further ongoing or unpublished studies.

##### Ongoing Trials

We will attempt to identify current and ongoing trials via this trial registry:

International Clinical Trials Registry Platform Search Portal

We will use this search statement:

Anxiety in the ‘Title’ AND Asperger OR Autism in the ‘Condition’.

#### Management of References

A full set of search results will be directly imported into an Endnote Library wherever possible or, if necessary, manually entered into the Endnote Library. The Endnote Library will provide a means to manage the full set of references and to identify duplicates.

### Data Collection

#### Selection of studies

The PRISMA flow chart illustrates the process for the selection of studies (see Figure 1).

**Figure 1:**
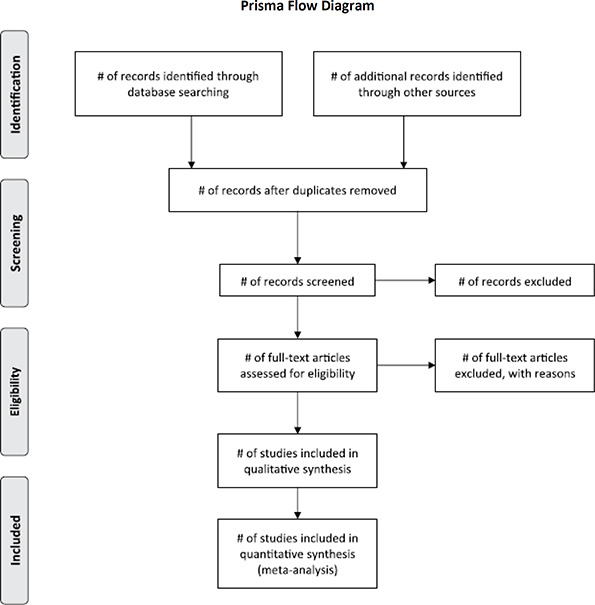
Process of study selection (Source: Moher et al., 2009)

As a first step in the screening process, all reviewers will independently assess titles and abstracts of a purposely heterogeneous subset of five studies identified through the searches. The purpose of this step is twofold: first it determines their potential eligibility for inclusion in the review and second it serves to develop a common understanding and application of inclusion criteria. Once a consensus regarding the application has been reached, all abstracts will be assessed by at least two reviewers. Where two reviewers disagree regarding the inclusion of an abstract in the study resolution will be sought through discussion or the decision of a third reviewer. At the end of this step, studies that clearly do not meet the criteria as well as duplicates will have been removed.

Full‐text articles will then be retrieved for the included abstracts. Data extraction from the full‐text articles will be undertaken independently by two reviewers. Extracted data will be compared between reviewers for seven articles. Any discrepancies will be resolved through discussion and, where necessary, further details added to data definitions which may be unclear. Data from a further two articles will be extracted to see whether agreement between reviewers has been reached.

Again, a selection of five articles will be critically appraised by all reviewers in order to ensure consistent application of criteria. As before, once consensus has been achieved, each article will be assessed independently by two reviewers for methodological validity using standardised critical appraisal instruments from the Joanna Briggs Institute Meta‐Analysis of Statistics Assessment and Review Instrument (JBI‐MAStARI) or the Qualitative Assessment and Review Instrument (JBI‐QARI). See Table 1 for a list of checklists and Appendix 1 for examples of the checklists. Any disagreements that arise between the reviewers will be resolved through discussion, or with a third reviewer. For studies that might reasonably have been expected to be included but which did not meet the inclusion criteria, the specific reasons for exclusion will be documented.

**Table 1:** The list of JBI critical appraisal tools that are available and may be used through their software in this review

• Checklist for Case Control Studies	(268 KB)
• Checklist for Case Reports	(263 KB)
• Checklist for Case Series	(279 KB)
• Checklist for Cohort Studies	(272 KB)
• Checklist for Diagnostic Test Accuracy Studies	(271 KB)
• Checklist for Economic Evaluations	(201 KB)
• Checklist for Prevalence Studies	(274 KB)
• Checklist for Quasi‐Experimental Studies (non‐randomized experimental)	(269 KB)
• Checklist for Randomized Controlled Trials	(269 KB)
• Checklist for Systematic Reviews	(278 KB)
• Checklist for Text and Opinion	(261 KB)
• Checklist for Analytical Cross Sectional Studies	(263 KB)
• Checklist for Qualitative Research	(187 KB)

#### Data extraction and management

Once papers are selected for inclusion in the review, data will be extracted using the standardised data extraction tools from JBI‐MAStARI and (JBI‐QARI). See Appendix 2 for the quantitative and qualitative data extraction forms. The data extracted from quantitative studies will include specific details about the interventions, populations, study methods and outcomes of significance to the review question and specific objectives. The data extracted from qualitative studies will include specific details about the phenomena of interest, populations, study methods and outcomes of significance to the review question and specific objectives.

While the forms provided in Appendix 2 are currently generic, information specific to this review ‐ for example the ASD diagnosis or which components of Rotheram‐Borus et al.'s (2012) framework for interventions were present – will be added after a discussion by the reviewer team prior to the start of the data extraction.

Where data are missing, authors will be contacted to obtain the relevant information. In some instances, it is possible to recreate missing data from available information. Where this is done, the assumptions and calculations will be detailed in the review report.

Only one outcome per study will be used in the separate quantitative and qualitative syntheses to avoid double counting. The primary outcome of a study will be chosen for data extraction. Where the primary outcome cannot be ascertained an outcome will be randomly chosen.

### Data Synthesis and Analysis

Quantitative data will, where possible, be pooled by way of statistical meta‐analysis using JBI‐MAStARI. All results will be subject to double data entry. Effect sizes expressed as odds ratio (for categorical data) and weighted mean differences (for continuous data) and their 95% confidence intervals will be calculated for analysis. Heterogeneity will be assessed statistically using the standard Chi‐square and also explored using subgroup analyses based on the different study designs included in this review. Where statistical pooling is not possible the findings will be presented in narrative form including tables and figures to aid in data presentation where appropriate.

Qualitative research findings will, where possible be pooled using JBI‐QARI. This will involve the aggregation or synthesis of findings to generate a set of statements that represent that aggregation, through assembling the findings rated according to their quality, and categorising these findings on the basis of similarity in meaning. These categories are then subjected to a meta‐synthesis in order to produce a single comprehensive set of synthesised findings that can be used as a basis for evidence‐based practice. Where textual pooling is not possible the findings will be presented in narrative form.

In the final step, the results of the quantitative review and the qualitative review will be integrated using the JBI Mixed Methods Aggregation Instrument (MMARI). To achieve integration, quantitative review findings will be translated into qualitative results through Bayesian conversion to produce synthesised findings.

#### Subgroup and heterogeneity analysis

In order to understand what interventions work for whom and how, we will investigate, where available, demographic factors such as gender, age, and setting. This may also inform the heterogeneity between interventions.

Heterogeneity analysis is used in meta‐analysis to estimate the degree to which the heterogeneity of effect sizes across studies is due to true heterogeneity or within‐study error (Borenstein et al., 2009). A general procedure in estimating heterogeneity is to compute the study‐to‐study observed variation, estimate the expected variation amount if the true effect were the same across the studies, and then calculate the remaining variation. We would assume this variation to represent true differences, or heterogeneity, in the effect size distribution. Rather than assessing whether heterogeneity is present, we will use the I^2^ statistic, reported as part of the Forest Plot available in JBI‐MAStARI software, to help assess the proportion of variability associated with between‐study heterogeneity. The I^2^ statistic represents the percentage variability in effect size estimates that is due to heterogeneity rather than sampling variability (Higgins & Green, 2011). The general guide of 50% is interpreted to represent moderate heterogeneity. In addition to reporting I^2^ as a relative measure of heterogeneity, the heterogeneity parameter estimate (τˆ2) as an absolute measure of heterogeneity will also be reported.

#### Testing for publication bias

Assessing risk of publication bias will be an important task because of its potential influence on estimates of intervention effects. This review will analyse and ameliorate possible publication bias by implementing the trim‐and‐fill method (Duval & Tweedie, 2000) using the Meta package in R (Schwarzer, 2007)^2^. This allows an initial assessment of whether unpublished data on Autism and anxiety interventions (likely to have null results) is an important issue in this area (Uljarević & Hamilton, 2013).

#### Sensitivity analysis

Given the diversity of interventions and the potentially small sample of included studies within each intervention type, it will be important to conduct a sensitivity analysis of the impact of a single study or the impact of including studies of varying levels of quality on the overall observed effect size for interventions in any meta‐analysis. Two main sensitivity analyses will be conducted, one excluding studies of lower quality and one focussing on excluding single studies which may have had an unduly large effect on the results. Results will then be compared to provide an indication of the robustness of the review's findings.

## TIMEFRAME

We anticipate the following updated timeline. Due to delays in the protocol review process, we have requested an extension for the submission of the draft final review to December 2017. This will delay the publication of the final review until February 2018 which we hope will be acceptable.

**Table:** 

**Phase**	**Stage**	**Timeframe**
Title registration	Title registration published	Sep 2016
Protocol development	Protocol preparation	Sep – Oct 2016
	Protocol draft submission	31 Oct 2016
	Protocol review	April‐September 2017
	Protocol acceptance	October 2017
Review	Study search	Sep 2017‐Mid Oct 2017
	Abstract screening	Mid‐End Oct 2017
	Document retrieval	Early Nov 2017
	Study eligibility screening	Mid Nov 2017
	Extraction of data and assessment of study quality/coding	Late Nov 2017
	Statistical analysis and qualitative data analysis	Early Dec 2017
	Preparation of draft review	Mid Dec 2017
	Submission of draft review	End Dec 2017
	Revision of draft review	Jan 2018
Publication	Publication of final review	Feb 2018

## REVIEW AUTHORS

**Lead review author:** The lead author will develop and co‐ordinates the review team, discuss and assign roles for individual members of the review team, liaise with the editorial base and take responsibility for the on‐going updates of the review.

**Table:** 

**Name:**	**Petra Lietz**
Title:	Dr
Affiliation:	Australian Council *for* Educational Research
Address:	186B Pulteney Street
City, State, Province or County:	Adelaide, South Australia
Postal Code:	5000
Country:	Australia
Phone:	(0)8‐8206 8611
Email:	petra.lietz@acer.edu.au
**Co‐authors**:	
**Name:**	**Julie Kos**
Title:	Dr
Affiliation:	Australian Council *for* Educational Research
Address:	19 Prospect Hill Road
City, State, Province or County:	Camberwell, Victoria
Postal Code:	3124
Country:	Australia
Phone:	(0)3‐9277 5420
Email:	julie.kos@acer.edu.au
	
**Name:**	**Elizabeth O’Grady**
Title:	Ms
Affiliation:	Australian Council *for* Educational Research
Address:	19 Prospect Hill Road
City, State, Province or County:	Camberwell, Victoria
Postal Code:	3124
Country:	Australia
Phone:	(0)3‐9277 5695
Email:	elizabeth.ogrady@acer.edu.au
Title:	Dr
Affiliation:	Olga Tennison Autism Research Centre, School of Psychology and Public Health, La Trobe University
Address:	Bundoora
City, State, Province or County:	Victoria
Postal Code:	3068
Country:	Australia
Phone:	(0)3‐94796762
Email:	m.uljarevic@latrobe.edu.au
	
**Name:**	**Katherine Dix**
Title:	Dr
Affiliation:	Australian Council *for* Educational Research
Address:	186B Pulteney Street
City, State, Province or County:	Adelaide, South Australia
Postal Code:	5000
Country:	Australia
Phone:	(0)8‐8206 8611
Email:	katherine.dix@acer.edu.au

## ROLES AND RESPONSIBILITIES

A brief description of content and methodological expertise within the review team is provided. It includes personnel on the review team who have content expertise, methodological expertise, statistical expertise, and information retrieval expertise.

### Content:

Dr Julie Kos will provide the content expertise. Dr Kos has published on the topic of Attention‐Deficit/Hyperactivity Disorder (ADHD). Published articles include Kos et al. (2006); Kos et al. (2004); Kos & Richdale (2004).

For further content guidance, she will work closely with the proposed advisory panel members, particularly Dr Mirko Uljarević who has agreed to contribute detailed topic relevant advice to the review free of charge in return for being a co‐author on the published review.

### Systematic review methods:

Dr Petra Lietz was a co‐author of systematic reviews (Best et al., 2013; Lietz et al., 2016) and meta‐analyses (Lietz 2006a, b) demonstrating her expertise with these methods.

Dr Katherine Dix has been formally trained and is an accredited JBI Comprehensive Systematic Review reviewer.

### Statistical analysis:

Dr Petra Lietz has extensive experience in statistical analyses as evidenced by her many publications, including: Lietz (2006a, b, c); Lietz & Kotte (2006); Schreier & Lietz (2006); Lietz & Kotte (2005).

### Information retrieval:

Ms Jenny Trevitt is an experienced librarian with more than 10 years' experience as a librarian in ACER's Cunningham Library. Ms Trevitt has also been directly involved with information retrieval for previous systematic reviews as detailed above.

### Support staff:

Mrs Elizabeth O'Grady will provide research support to the review team. Elizabeth has extensive experience in education research methods as well as statistical analysis as demonstrated by the following publications: Redmond et al. (2016); Lietz et al. (2015); De Bortoli et al. (2014).

### Advisory panel:

The following members of the advisory panel will provide invaluable expertise to guide the proposed review. A brief bio statement for each is provided in the Registration of Title.

- Professor Cheryl Dissanayake
- Dr Mirko Uljarević
- Dr Giacomo Vivanti
- Associate Professor Amanda Richdale
- Dr Darren Hedley

## PLANS FOR UPDATING THE REVIEW

Petra Lietz, as lead author, will be responsible for updates of the review.

## ACKNOWLEDGEMENTS

We would like to thank the support of The Joanna Briggs Institute.

## FUNDING

Neither the team members who are proposing to undertake this systematic review nor the Australian Council for Educational Research receive any financial support from any other source for this project, nor do they intend to apply for funding from other sources.

## POTENTIAL CONFLICTS OF INTEREST

There are no known potential conflicts of interest.

## AUTHOR DECLARATION

**Authors' responsibilities**

By completing this form, you accept responsibility for preparing, maintaining, and updating the review in accordance with Campbell Collaboration policy. The Coordinating Group will provide as much support as possible to assist with the preparation of the review.

A draft protocol must be submitted to the Coordinating Group within one year of title acceptance. If drafts are not submitted before the agreed deadlines, or if we are unable to contact you for an extended period, the Coordinating Group has the right to de‐register the title or transfer the title to alternative authors. The Coordinating Group also has the right to de‐register or transfer the title if it does not meet the standards of the Coordinating Group and/or the Campbell Collaboration.

You accept responsibility for maintaining the review in light of new evidence, comments and criticisms, and other developments, and updating the review every five years, when substantial new evidence becomes available, or, if requested, transferring responsibility for maintaining the review to others as agreed with the Coordinating Group.

**Publication in the Campbell Library**

The support of the Coordinating Group in preparing your review is conditional upon your agreement to publish the protocol, finished review, and subsequent updates in the Campbell Library. The Campbell Collaboration places no restrictions on publication of the findings of a Campbell systematic review in a more abbreviated form as a journal article either before or after the publication of the monograph version in *Campbell Systematic Reviews*. Some journals, however, have restrictions that preclude publication of findings that have been, or will be, reported elsewhere and authors considering publication in such a journal should be aware of possible conflict with publication of the monograph version in *Campbell Systematic Reviews*. Publication in a journal after publication or in press status in *Campbell Systematic Reviews* should acknowledge the Campbell version and include a citation to it. Note that systematic reviews published in *Campbell Systematic Reviews* and co‐registered with the Cochrane Collaboration may have additional requirements or restrictions for co‐publication. Review authors accept responsibility for meeting any co‐publication requirements.

I understand the commitment required to undertake a Campbell review, and agree to publish in the Campbell Library. Signed on behalf of the authors:

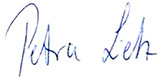

**Table:** 

Form completed by: Dr Petra Lietz	**Date:** 31 October 2016

## Appendix

Source:

http://joannabriggs.org/assets/docs/critical‐appraisal‐tools/JBI_Critical_Appraisal‐Checklist_for_Randomized_Controlled_Trials.pdf

Source:

http://joannabriggs.org/assets/docs/critical‐appraisal‐tools/JBI_Critical_Appraisal‐Checklist_for_Quasi_‐_Experimental_Studies.pdf

Source:

http://joannabriggs.org/assets/docs/critical‐appraisal‐tools/JBI_Critical_Appraisal‐Checklist_for_Qualitative_Research.pdf

## Appendix

**Table:**

**Academic Research Complete (EBSCO)**	
**Search Sets**	**Search Queries**
	Search Mode: Boolean/Phrase. Related Terms Not Applied.
	Date Range: 1996‐2017
S7	S1 AND S2 AND S5 AND S6
S6	Intervention OR treatment OR therap* OR psychotherap*
S5	S3 OR S4
S4	SU (children OR adolescence OR youth OR teenagers OR boys OR girls) OR (young people OR young person)
S3	SU students AND SU school*
S2	SU (anxiety OR fear) OR (anxious OR internal*)
S1	SU (AUTIS* OR ASPERGER’S syndrome) OR (ASD OR Pervasive Developmental Disorder OR PDD NOS OR PDD unspecified)

**Table:**

**A+Education (Informit)**	
**Search Sets**	**Search Queries**
	Date Range: 1996‐2017
s3	#1 AND #2
S2	Anxiety OR anxious OR internali* OR fear Includes Subject Terms: Anxiety Fear
S1	ASD OR Asperger* OR autis* OR “Pervasive Developmental” OR “PDD NOS” OR “PDD unspecified” Includes Subject Terms: Autism spectrum disorders Asperger syndrome

**Table:**

**British Education Index (EBSCO)**	
**Search Sets**	**Search Queries**
	Search Mode: Boolean/Phrase. Related Terms Not Applied.
	Date Range: 1996‐2017
S7	S15 AND S16 AND S19 AND S20
S6	Intervention OR treatment OR therap* OR psychotherap* OR evaluation OR outcome OR program OR trial* OR experimental OR control group OR random* OR best practi* or evidence
S5	S3 OR S4
S4	SU students AND SU school*
S3	child* OR adolescen* OR preadolescen* OR pre adolescen* OR youth OR teen* OR teen age* OR young people OR young person OR boy OR girl
S2	Anxiety OR anxious OR internali* OR fear Includes Subject Terms: Anxiety Fear
S1	ASD OR Asperger* OR autis* OR Pervasive Developmental Disorder OR PDD NOS OR PDD unspecified Includes Subject Terms: AUTISM AUTISM spectrum disorders ASPERGER'S syndrome AUTISM in children AUTISTIC children Autism in adolescence PERVASIVE developmental disorder not otherwise specified

**Table:**

**CBCA Complete (Proquest)**	
**Search Sets**	**Search Queries**
	Date Range: 1996‐2017
S5	S1 AND S2 AND S3 AND S4
S4	all(Intervention) OR all(treatment) OR all(therap*) OR all(psychotherap*) OR all(evaluation) OR all(outcome) OR all(program) OR all(trial*) OR all(experimental) OR all(control group) OR all(random*) OR all(best practi*) or all(evidence based)
S3	all(Student OR child* OR adolescen* OR preadolescen* OR pre‐adolescen* OR youth OR teen* OR teen age* OR young people OR young person OR boy OR girl)
S2	(all(Anxiet*) OR all(anxious) OR all(internali*) OR all(fear)) Includes Subject Terms: Anxieties Fear & Phobias
S1	all(ASD OR Asperger* OR autis* OR Pervasive Developmental Disorder OR PDD NOS OR PDD unspecified) Includes Subject Terms: Autism Asperger syndrome

**Table:**

**CINAHL Plus with Full Text (EBSCO)**	
**Search Sets**	**Search Queries**
	Search Mode: Boolean/Phrase. Related Terms Not Applied.
	Date Range: 1996‐2017
S5	S1 AND S2 AND S3 AND S4
S4	Intervention OR treatment OR therap* OR psychotherap* OR evaluation OR outcome OR program OR trial* OR experimental OR control group OR random* OR best practi* or evidence based
S3	Student OR child* OR adolescen* OR preadolescen* OR pre adolescen* OR youth OR teen* OR teen age* OR young people OR young person OR boy OR girl AND Age Groups: Child, Preschool: 2‐5 years, Child: 6‐12 years, Adolescent: 13‐18 years, All Child
S2	SU (Anxiety OR Fear OR Anxiety Disorders) OR (Anxious* OR internali*)
S1	SU autistic disorder OR (ASD OR Asperger* OR autis* OR Pervasive Developmental Disorder OR PDD NOS OR PDD unspecified) Includes Subject Term: Pervasive Developmental Disorder‐Not Otherwise Specified

**Table:**

**Education Research Complete (EBSCO)**	
**Search Sets**	**Search Queries**
	Search Mode: Boolean/Phrase. Related Terms Not Applied.
	Date Range: 1996‐2017
	
S5	S1 AND S2 AND S3 AND S4
S4	Intervention OR treatment OR therap* OR psychotherap* OR evaluation OR outcome OR program OR trial* OR experimental OR control group OR random* OR best practi* or evidence based
S3	(SU students AND SU school*) OR (child* OR adolescen* OR preadolescen* OR pre adolescen* OR youth OR teen* OR teen age* OR preteen OR young people OR young person OR boy OR girl)
S2	SU (Anxiety OR Fear) OR (anxious OR internali*)
S1	SU (Autis* OR ASPERGER'S syndrome) OR (ASD OR Pervasive Developmental Disorder OR PDD NOS OR PDD unspecified)

**Table:**

**EMBASE (Elsevier)**	
**Search Sets**	**Search Queries**
	Date Range: 1996‐2017
S7	S6 AND ([adolescent]/lim OR [child]/lim OR [preschool]/lim OR [school]/lim OR [young adult]/lim)
S6	S1 AND S4 AND S5
S5	‘intervention’/exp OR ‘treatment’/exp OR ‘therapy’/exp OR ‘psychotherapy’/
S4	S2 OR S3
S3	internalis* OR internaliz*
S2	‘anxiety’/de OR ‘anxiety disorder’/de OR ‘fear’/
S1	‘autism’/de OR ‘asperger syndrome’/de OR ‘pervasive developmental disorder not otherwise specified’/de

**Table:**

**ERIC (EBSCO)**	
**Search Sets**	**Search Queries**
	Search Mode: Boolean/Phrase. Related Terms Not Applied.
	Date Range: 1996‐2017
S7	S1 AND S2 AND S3 AND S6
S6	S4 OR S5
	(Student OR child* OR adolescen* OR preadolescen* OR pre adolescen* OR youth OR teen* OR teen age* OR young people OR young person OR boy OR girl) AND
S5	Educational Level: Early Childhood Education, Elementary Education, Elementary Secondary Education, Grade 1, Grade 2, Grade 3, Grade 4, Grade 5, Grade 6, Grade 7, Grade 8, Grade 9, Grade 10, Grade 11, Grade 12, High School Equivalency Programs, High Schools, Intermediate Grades, Junior High Schools, Kindergarten, Middle Schools, Preschool Education, Primary Education, Secondary Education
S4	DE (Children OR Young Children OR Adolescents OR Preadolescents OR Youth OR High School Students OR Elementary School Students OR Middle School Students OR Secondary School Students OR Junior High School Students)
S3	(Intervention OR treatment OR therap* OR psychotherap* OR evaluation OR outcome OR program* OR trial* OR experimental OR quasiexperimental OR control group OR random* OR Best practi* or evidence based)
S2	(Anxiety OR anxious OR internali* OR fear) Includes Subject Terms: Anxiety Fear Anxiety Disorders
S1	(ASD OR Asperger* OR autis* OR Pervasive Developmental Disorder OR PDD NOS OR PDD unspecified) Includes Subject Terms: Autism Pervasive Developmental Disorders Asperger Syndrome

**Table:**

**PsycInfo (EBSCO)**	
**Search Sets**	**Search Queries**
	Search Mode: Boolean/Phrase. Related Terms Not Applied.
	Date Range: 1996‐2017
S7	S1 AND S2 AND S5 AND S6
S6	S3 OR S4
S5	SU (Intervention OR treatment OR therap* OR psychotherap* OR evaluation OR outcomes OR program OR experimental OR “experiment controls” OR “random sampling” OR “best practices” or “evidence based practices”) OR TI (Intervention OR treatment OR therap* OR psychotherap*) OR AB (Intervention OR treatment OR therap* OR psychotherap*)
S4	(student OR child* OR adolescen* OR preadolescen* OR pre adolescen* OR youth OR teen* OR teen age* OR young people OR young person OR boy OR girl) AND Age Groups: Childhood (birth‐12 yrs), Preschool Age (2‐5 yrs), S chool Age (6‐12 yrs), Adolescence (13‐17 yrs), Young Adulthood (18‐29 yrs)
S3	DE Kindergarten Students OR Preschool Students OR Elementary School Students OR Intermediate School Students OR Primary School Students OR Middle School Students OR High School Students OR Junior High School Students
S2	SU (Anxiety OR Fear OR Internalization) OR TI (anxious OR internali*) OR AB (anxious OR internali*)
S1	DE Autism Spectrum Disorders OR TI (ASD OR Asperger* OR autis* OR Pervasive Developmental Disorder OR PDD NOS OR PDD unspecified) OR AB (ASD OR Asperger* OR autis* OR Pervasive Developmental Disorder OR PDD NOS OR PDD unspecified)

**Table:**

**PubMed**	
**Search Sets**	**Search Queries**
	Date Range: 1996‐2017
S10	(S9 AND S8 AND S4AND S3)
S9	(therapeutics[Text Word] OR intervention[Text Word] OR therapy[Text Word] OR psychotherapy[Text Word] OR treatment[Text Word])
S8	(S5 OR S6 OR S7)
S7	(child*[Title/Abstract] OR adolescen*[Title/Abstract] OR preadolescen*[Title/Abstract] OR pre adolescen*[Title/Abstract] OR youth[Title/Abstract] OR teen*[Title/Abstract] OR teen age*[Title/Abstract] OR young people[Title/Abstract] OR young person[Title/Abstract] OR girl*[Title/Abstract] OR boy*[Title/Abstract])
S6	(STUDENT*[Title/Abstract]) AND SCHOOL*[Title/Abstract]
S5	(“adolescent”[MeSH Terms]) OR “child”[MeSH Terms]
S4	(“anxiety”[MeSH Terms] OR “anxiety disorders”[MeSH Terms] OR “fear”[MeSH Terms]) OR (internaliz*[Title/Abstract] OR internalis*[Title/Abstract] OR anxious[Title/Abstract] OR anxiety[Title/Abstract] OR fear[Title/Abstract])
S3	(S1 OR S2)
S2	(ASD[Title/Abstract] OR autism[Title/Abstract] OR autistic[Title/Abstract] OR Asperger*[Title/Abstract] OR “PDD NOS”[Title/Abstract] OR “PDD unspecified”[Title/Abstract])
S1	“child development disorders, pervasive”[MeSH Terms]

**Table:**

**SCOPUS (Elsevier)**	
**Search**	**Search Queries**
**Set**	No Thesaurus available. Date Range: 1996‐2017
S5	S1 AND S2 AND S3 AND S4 AND Collection Limiters: Social Sciences Arts & Humanities Neuroscience
S4	TITLE‐ABS‐KEY (intervention OR treatment OR therap* OR psychotherap* OR evaluation OR outcome OR program* OR trial* OR experimental OR (control W/0 group) OR random* OR (best W/0 practi*) OR “evidence based”)
S3	TITLE‐ABS‐KEY (student OR child* OR adolescen* OR preadolescen* OR (pre W/0 adolescen*) OR youth OR teen* OR (teen W/0 age*) OR “young people” OR “young person” OR boy OR girl)
S2	TITLE‐ABS‐KEY (anxiety OR anxious OR internali* OR fear)
S1	TITLE‐ABS‐KEY (asd OR asperger* OR autis* OR “Pervasive Developmental” W/0 disorder* OR “PDD NOS” OR “PDD unspecified)

**Table:**

**SocIndex (EBSCO)**	
**Search Terms**	**Search Queries**
	Search Mode: Boolean/Phrase. Related Terms Not Applied.
	Date Range: 1996‐2017
**S5**	(S1 AND S2 AND S3 AND S4)
**S4**	Intervention OR treatment OR therap* OR psychotherap* OR evaluation OR outcome OR program OR trial* OR experimental OR control group OR random* OR best practi* or evidence based
**S3**	Student OR child* OR adolescen* OR preadolescen* OR pre adolescen* OR youth OR teen* OR teen age* OR young people OR young person OR boy OR girl
**S2**	Anxiety OR anxious OR internali* OR fear
**S1**	ASD OR Asperger* OR autis* OR Pervasive Developmental Disorder OR PDD NOS OR PDD unspecified
